# Role of Kuroshio Current in fish resource variability off southwest Japan

**DOI:** 10.1038/s41598-019-54432-3

**Published:** 2019-11-29

**Authors:** Yushi Morioka, Sergey Varlamov, Yasumasa Miyazawa

**Affiliations:** Application Laboratory, VAiG, JAMSTEC, Yokohama, Japan

**Keywords:** Marine biology, Physical oceanography

## Abstract

Western boundary currents in the subtropics play a pivotal role in transporting warm water from the tropics that contribute to development of highly diverse marine ecosystem in the coastal regions. As one of the western boundary currents in the North Pacific, the Kuroshio Current (hereafter the Kuroshio) exerts great influences on biological resource variability off southwest Japan, but few studies have examined physical processes that attribute the coastal fish resource variability to the basin-scale Kuroshio variability. Using the high-quality fish catch data and high-resolution ocean reanalysis results, this study identifies statistical links of interannual fish resource variability off Sukumo Bay, Shikoku island of Japan, to subsurface ocean temperature variability in the Kuroshio. The subsurface ocean temperature variability off the south of Sukumo Bay exhibits vertically coherent structure with sea-surface height variability, which originates from the westward-propagating oceanic Rossby waves generated through surface wind anomalies in the Northwest Pacific. Although potential sources of the atmospheric variability remain unclarified, the remotely-induced oceanic Rossby waves contribute to fish resource variability off Sukumo Bay. These findings have potential applications to other coastal regions along the western boundary currents in the subtropics where the westward-propagating oceanic Rossby waves may contribute to coastal ocean temperature variability.

## Introduction

Western boundary currents in the subtropics contribute to the establishment of rich marine ecosystem along the coastal regions through poleward transport of warm water from the tropics^1^. The warm water along the western boundary currents also plays a crucial role in the formation of a relatively warm and moist climate in the western side of ocean basin^2,3^. One of the western boundary currents in the subtropical North Pacific, called the Kuroshio Current (hereafter the Kuroshio), provides favorable environment for highly diverse marine ecosystems off Philippines, Taiwan, and the south of Japan^4–6^. Since these countries are traditionally dependent on fish resources from the Kuroshio as sources of protein foods, understanding physical processes in the Kuroshio and its potential link with fish resource variability is greatly important.

The Kuroshio variability has been extensively studied across different temporal and spatial scales using observation data and modelling techniques^7–9^. In particular, off the south of Japan, the Kuroshio frequently undergoes small and large meanderings in a meridional orientation^10–15^, which have great influences on spatial distribution of fish species^4,16^. Although there are several physical processes proposed to explain the meridional fluctuations of the Kuroshio^11,17–22^, westward-propagating oceanic Rossby waves from the Northwest Pacific associated with ocean density variations are considered to play an important role in triggering small meanderings off Kyushu island in southwest Japan^19,21–23^. This variation may further cause small meanders off the southwest of Shikoku island^24^ and large meanders off Kii Peninsula^13,17^ on interannual timescales. However, most of the previous studies limit their discussions to the underlying physical processes behind the Kuroshio variability, and potential impacts on the amount of fish resources off the south of Japan have been poorly understood.

One of the coastal bays located off southwest Japan, Sukumo Bay, is known to be greatly influenced by the Kuroshio variability^25,26^. Climatologically, the Kuroshio off Kyushu island first approaches south of Sukumo Bay, then turns eastward along the south coast of Japan. The northward approach of the Kuroshio brings warm water into Sukumo Bay, and the interaction with nutrient-rich cold water from Bungo Channel provides favorable conditions for biological activity off Sukumo Bay. Physical processes and predictability of warm water intrusion in Sukumo Bay are well-examined in the previous studies^25–29^, but the potential influence on the fish resource variability has yet to be investigated in the context of the links with basin-scale Kuroshio variability.

To bridge a gap in our current understanding of the relationship between coastal fish resources and basin-scale ocean current variability, this study aims to (i) establish statistical links of the Kuroshio variability to the fish resources off Sukumo Bay and (ii) identify potential sources of the Kuroshio variability. For these purposes, we utilize high-resolution ocean reanalysis results in the Northwest Pacific with the ability to resolve the Kuroshio variability involving mesoscale eddies. Also, high-quality fish catch data with daily catch efforts off Sukumo Bay are used to estimate monthly fish resources and their variability. The identified relationship would be beneficial for establishing fish resource prediction and management off Sukumo Bay, and have potential applications to other coastal regions along the western boundary currents in the subtropics.

## Results

### Impact of local processes on fish resource variability

**Figure 1:**
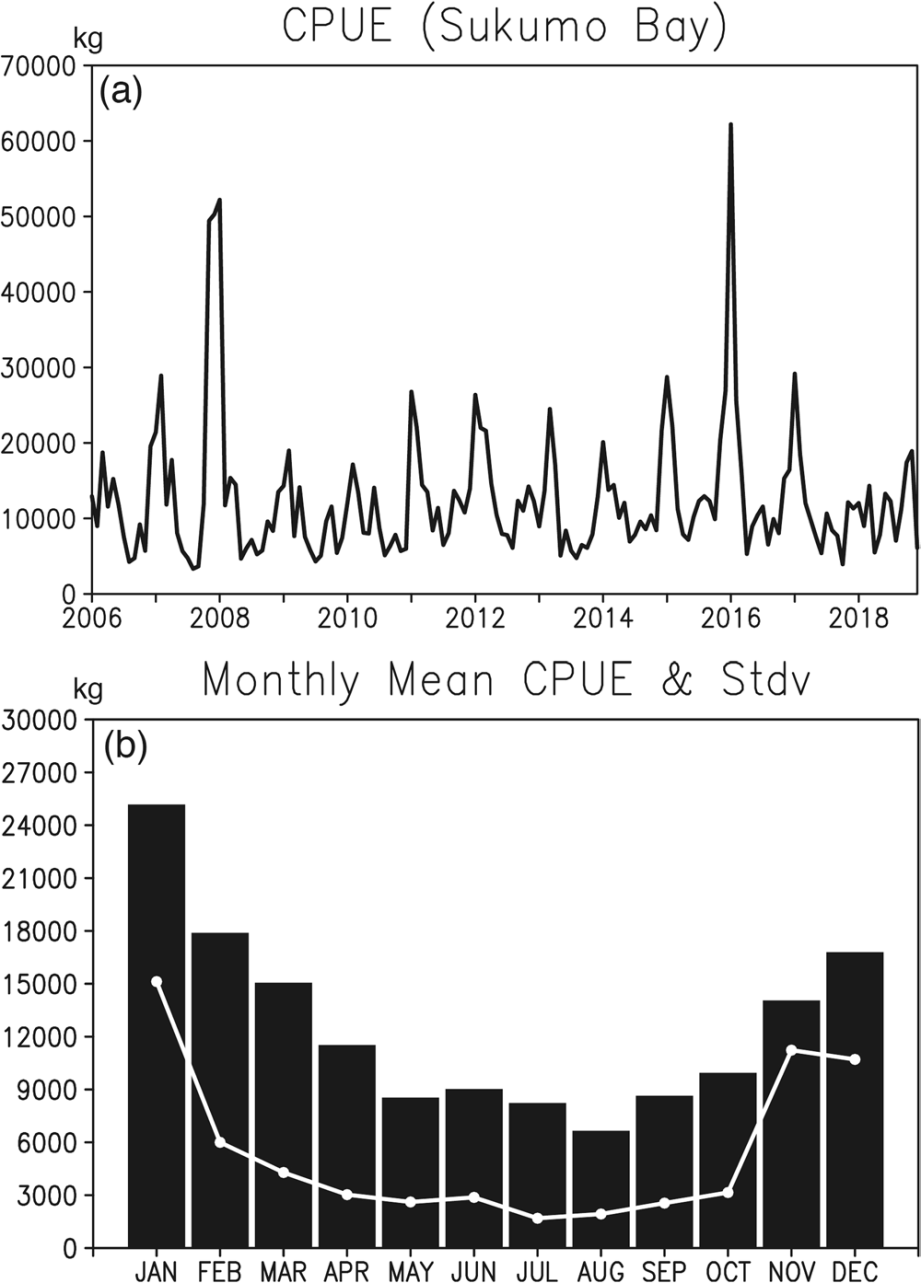
(**a**) Monthly fish catch per unit effort (CPUE, in kg) based on mid-size surrounding nets off Sukumo Bay during 2006–2018. (**b**) Monthly climatology of CPUE (bar, in kg) and its standard deviation (solid white line, in kg).

Fish resources off Sukumo Bay undergo pronounced seasonal to interannual variability. Monthly fish catch per unit effort (CPUE) during 2006–2018 exhibits remarkable year-to-year variations with high values in 2007 and 2015 and low values in 2009 and 2010 (Fig. 1a). The interannual variability of the CPUE off Sukumo Bay is found to be due mostly to the wintertime variability. This can be clearly seen in Fig. 1b showing a strong seasonality of the CPUE with the large standard deviation during November-January. Further analysis of fish species data also shows that during boreal winter, small fishes such as sardine and horse mackerel become the dominant species off Sukumo Bay due probably to the combined effects of the nutrient-rich southward current from Bungo Channel and the northward warm water intrusion of the Kuroshio. Given that most of the annual CPUE is explained by the wintertime catches, this study focuses on identifying physical processes that control interannual variability in the wintertime CPUE off Sukumo Bay.

**Figure 2:**
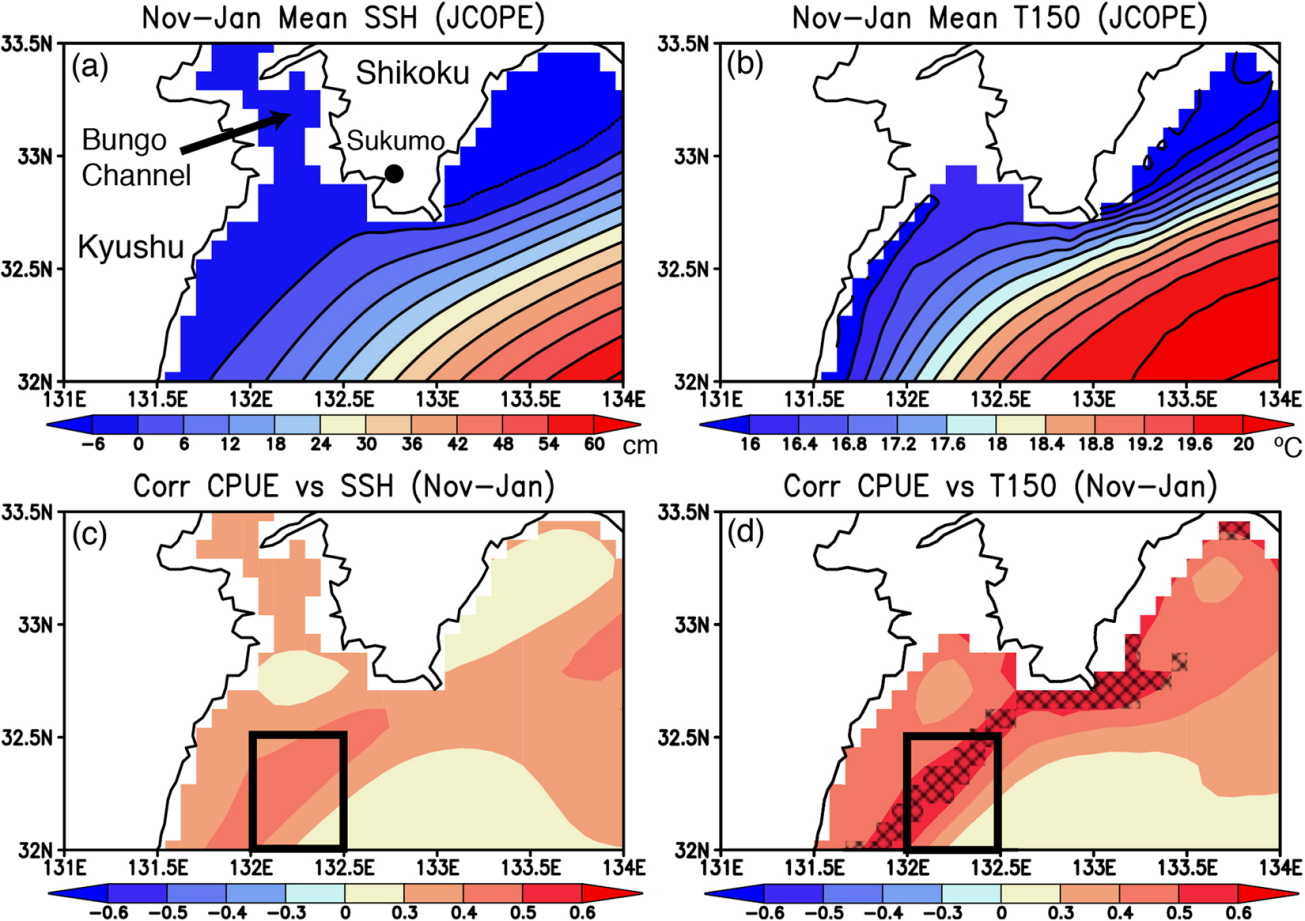
(**a**) November-January mean of sea-surface height (SSH, in cm) from the JCOPE reanalysis results. (**b**) Same as in (**a**), but for the subsurface ocean temperature (in °C) at 150 m depth. (**c**) Correlation coefficient between November-January mean CPUE and SSH. A black box indicates our region of interest off the south of Sukumo Bay. (**d**) Same as in (**c**), but for the correlation coefficient between November-January mean CPUE and subsurface ocean temperature at 150 m depth. Hatched areas indicate correlation coefficients which are statistically significant above 90% confidence level of a Student *t*-test.

Spatial patterns of November-January mean sea-surface height (SSH) and subsurface ocean temperature at 150 m depth (T150) are presented in Fig. 2a,b. The Kuroshio, the core of which is estimated by the strongest gradient of the SSH, flows northeastward off Shikoku island. The spatial distribution of T150 mostly follows a similar pathway of the Kuroshio, although some coastal region (132–132.5°E and 32–32.5°N) on the western flank of the Kuroshio shows relatively warm temperature, representing northward intrusion of warm water from the Kuroshio to Bungo Channel. This region well corresponds to the south of major fishing areas during boreal winter.

The wintertime CPUE off Sukumo Bay shows moderately high correlation (0.4–0.5) with the SSH and T150 off the south of Sukumo Bay (Fig. 2c,d). Here we calculated the correlation between the November-January mean SSH/T150 data from 2006 to 2018 (i.e. 13 values) as two-dimensional (i.e. latitude/longitude) oceanic variables from the JCOPE reanalysis and the November-January mean one-dimensional CPUE data after aggregating all the CPUE data reported in different regions off the Sukumo Bay. Assuming that the one-dimensional CPUE data represents the area-averaged fish resource off the Sukumo Bay, we constructed the two-dimensional correlation coefficients between the CPUE and the SSH/T150 over the grid cells of the JCOPE model, so the estimated correlation coefficients have 11 degrees of freedom. It should also be noted that lag autocorrelation of November-January CPUE shows a very weak relation with the CPUE beyond two months (Fig. S1), so the wintertime CPUE variability is not much affected by other seasons’ CPUE variability. Although some region near the southwestern tip of Shikoku island shows relatively high correlation, both the SSH and the T150 off the south of Sukumo Bay (132–132.5°E and 32–32.5°N) show a coherent structure with moderately high correlations along the Kuroshio. In particular, the correlation coefficient with the T150 is statistically significant at 90% confidence level, although that with the SSH is not significant. Even if the outlier year of 2016 associated with extremely high CPUE (Fig. 1a) is removed from the analysis, the correlation values with the SSH and T150 remain relatively high above 0.4 off the south of Sukumo Bay (Fig. S2a,b), although the correlation coefficient with the T150 is statistically significant at 80% due to a decrease in the number of available data. This significant relationship with the T150 suggests that the above-normal subsurface ocean temperature associated with the northward approach of the Kuroshio may play an important role in providing favorable conditions for increase in the wintertime fish resources off Sukumo Bay. Since there is no clear relationship between the wintertime CPUE and the area-averaged Chlorophyll-a off the south of Sukumo Bay (Fig. S3), subsurface ocean temperature variations may directly contribute to changes in spatial distribution of small fishes off Sukumo Bay.

### Remote influences on Kuroshio variability

**Figure 3:**
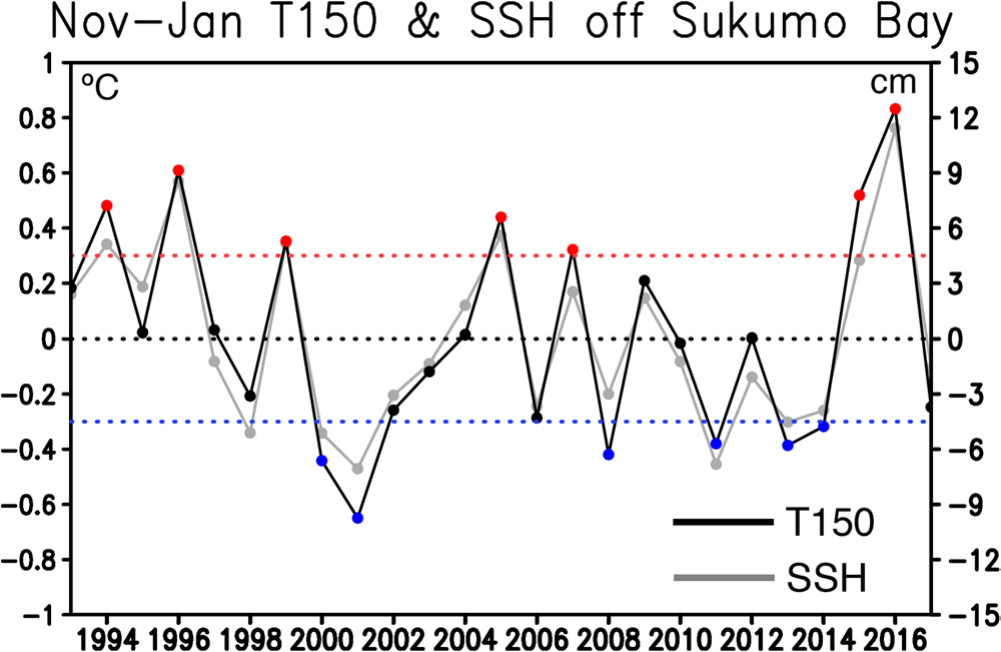
November-January mean subsurface ocean temperature anomalies at 150 m depth (black solid line, in °C) and SSH (grey solid line, in cm) anomalies during 1993–2018. Red and blue dotted lines exhibit one and negative one standard deviation of subsurface ocean temperature anomalies, respectively. Red and blue filled circles on the solid black line correspond to positive and negative events with anomalously high and low subsurface ocean temperature above one and negative one standard deviation, respectively.

Subsurface ocean temperature variability off Sukumo Bay shows strong association to the overlying SSH variability. This is evident in Fig. 3 showing time series of the T150 and SSH anomalies during boreal winter (November-January). It should be noted that we extend the time series back to 1993 when the satellite SSH data become available and incorporated into the ocean reanalysis results. The T150 anomalies remarkably fluctuate year by year, and exhibit a distinct in-phase relationship with the SSH anomalies. To investigate a physical link with the SSH variability and its potential sources, we define positive and negative events as years when the T150 anomalies during boreal winter exceed above one standard deviation and below negative one standard deviation, respectively. This leads to seven positive and six negative events, including higher than normal CPUE years (2007, 2015, and 2016) and lower than normal CPUE years (2013) in Fig. 1a. Since the negative events include the limited number of lower-than-normal CPUE year, we focus on the analysis of physical processes for the positive events.

The in-phase relationship with the SSH variability is found for subsurface ocean temperature at different depths. During positive events, the ocean temperature in the upper 700 m shows a warmer-than-average condition compared to that in all the analysis years (Fig. S4a). Since the ocean density becomes lower than that in all the analysis years but does not show much difference below 700 m (Fig. S4b), the thermocline located at around 200–300 m, defined as the maximum vertical gradient of ocean density below the surface mixed-layer, appears to become deeper. The associated downwelling induces above-normal subsurface ocean temperature through moving the isotherm layers downward. A similar but opposite relationship can be seen for the negative events. The strong coherence between the SSH and subsurface ocean temperature involving the thermocline variability is also reported in the observational studies over the Kuroshio recirculation region^30^.

**Figure 4:**
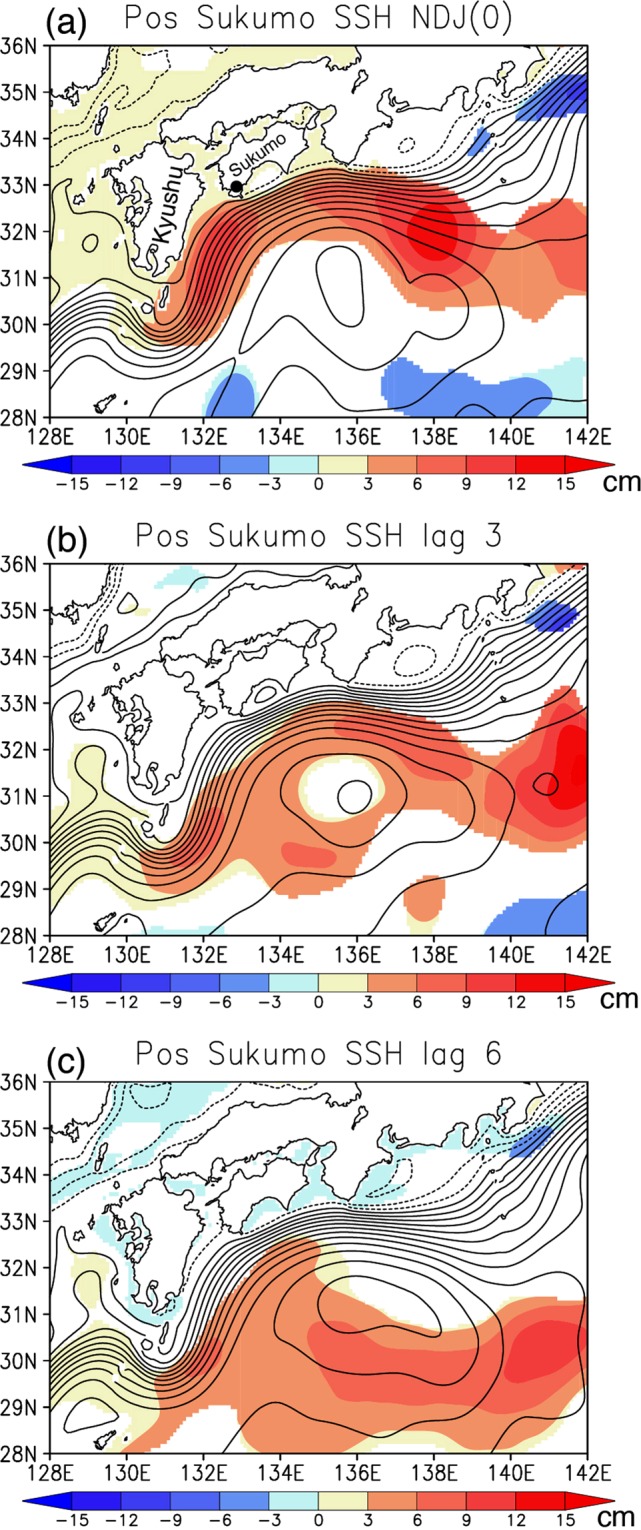
Lag composites of absolute SSH (contour, interval of 7 cm) and its anomalies (color, in cm) during the positive events. Seasonal mean anomalies for (**a**) November-January (NDJ(0)), (**b**) three-month lag (i.e. August-October), and (**c**) six-month lag (i.e. May-July) are shown, respectively. The black dot in (**a**) indicates Sukumo City. Statistically significant anomalies exceeding 90% confidence level using a Student *t*-test are colored.

To obtain useful insights into the generation of the SSH anomalies, spatial patterns of the SSH anomalies composited with every three-month lag during the positive events are presented in Fig. 4. The positive SSH anomalies off the south of Sukumo Bay during boreal winter (November-January; NDJ (0)) exhibit strong association with positive SSH anomalies along the Kuroshio pathway. In particular, the positive SSH anomalies off the south of Sukumo Bay appear to be a part of positive SSH anomalies east of Kyushu island. These anomalies seem to originate from the positive SSH anomalies to the southeast at three-month lag (132–136°E and 29–31°N in Fig. 4b) and further east at six-month lag (138–142°E and 29–31°N in Fig. 4c). On the other hand, there is very weak contribution of the SSH anomalies off the southwest Kyushu island at these lags. This lag-composite analysis suggests that most of the positive SSH anomalies off the south of Sukumo Bay may be related to westward migration of the positive SSH anomalies from the Northwest Pacific.

**Figure 5:**
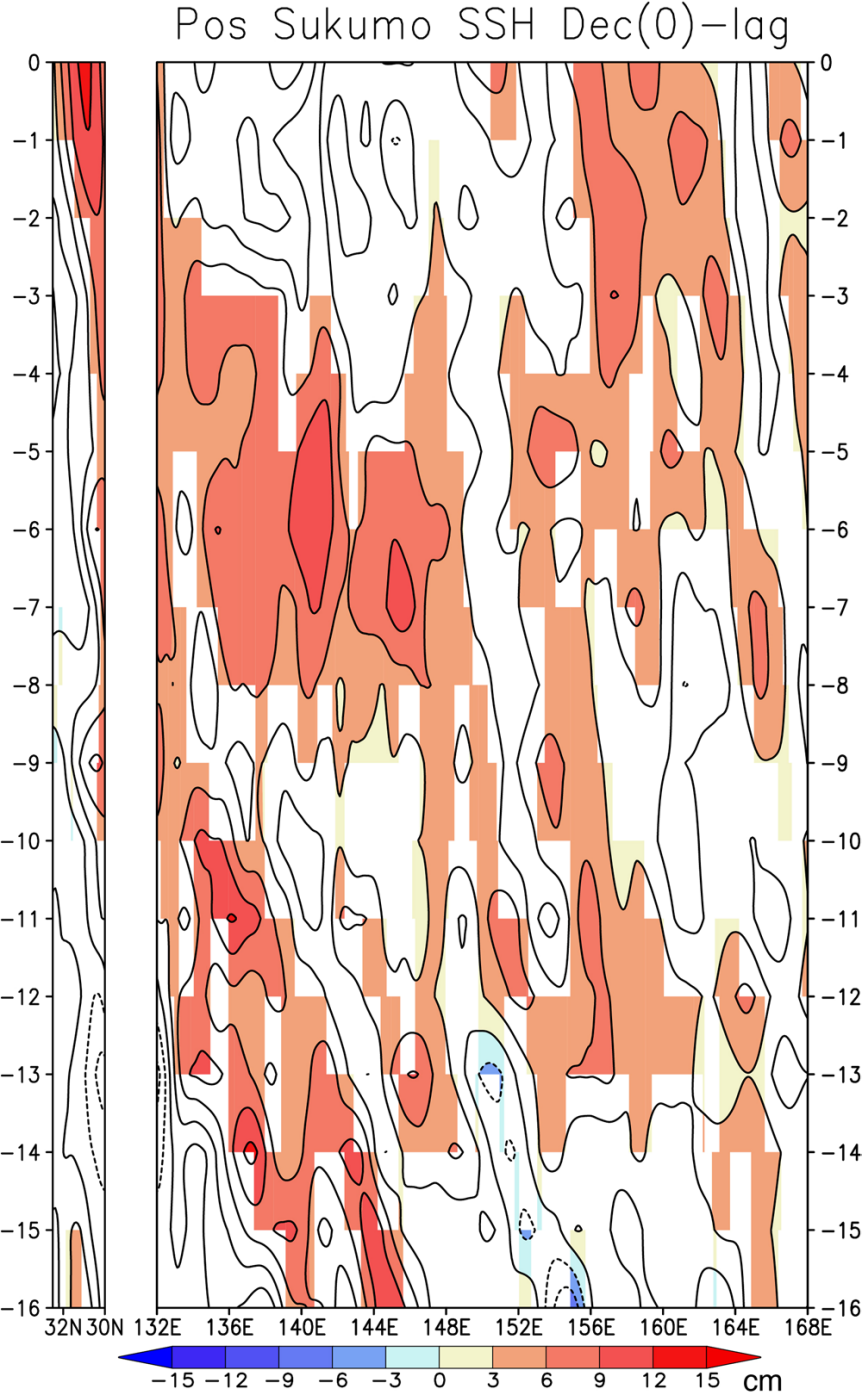
Monthly lag composites (16-month lag to 0-month lag, i.e. Dec (0)) of SSH anomalies during the positive events. The left panel shows north-south SSH anomalies (32.5–30°N) along 132°E, while the right panel shows west-east SSH anomalies (132–168°E) along 30°N. Statistically significant anomalies exceeding 90% confidence level using a Student *t*-test are shaded.

To highlight the migration of the SSH anomalies from the Northwest Pacific, we plot two Hovmöller diagrams for north-south SSH anomalies along 132°E and west-east SSH anomalies along 30°N as a function of time lag (Fig. 5). The positive SSH anomalies at zero-month lag (i.e. Dec (0)) off the south of Sukumo Bay are associated with clear northward propagation of the anomalies with two-month lag (left panel in Fig. 5). This indicates that the positive SSH anomalies migrate northward along the east coast of Kyushu island under the influence of the northward Kuroshio (Fig. 4a). The positive SSH anomalies further originate from the westward-propagating SSH anomalies with 12-month lag in the region of 150–160°E at 30°N (right panel in Fig. 5). The westward propagation speed is estimated to be around 10 cm s^−1^. This is higher, by a factor of two, than the theoretical phase speed (around 5 cm s^−1^) for the first baroclinic Rossby waves at 30°N, probably due to the acceleration of the oceanic Rossby waves interacting with the mean current field^31^. As such, the oceanic Rossby waves generated in the Northwest Pacific may contribute to the generation of the positive SSH anomalies off Sukumo Bay.

**Figure 6:**
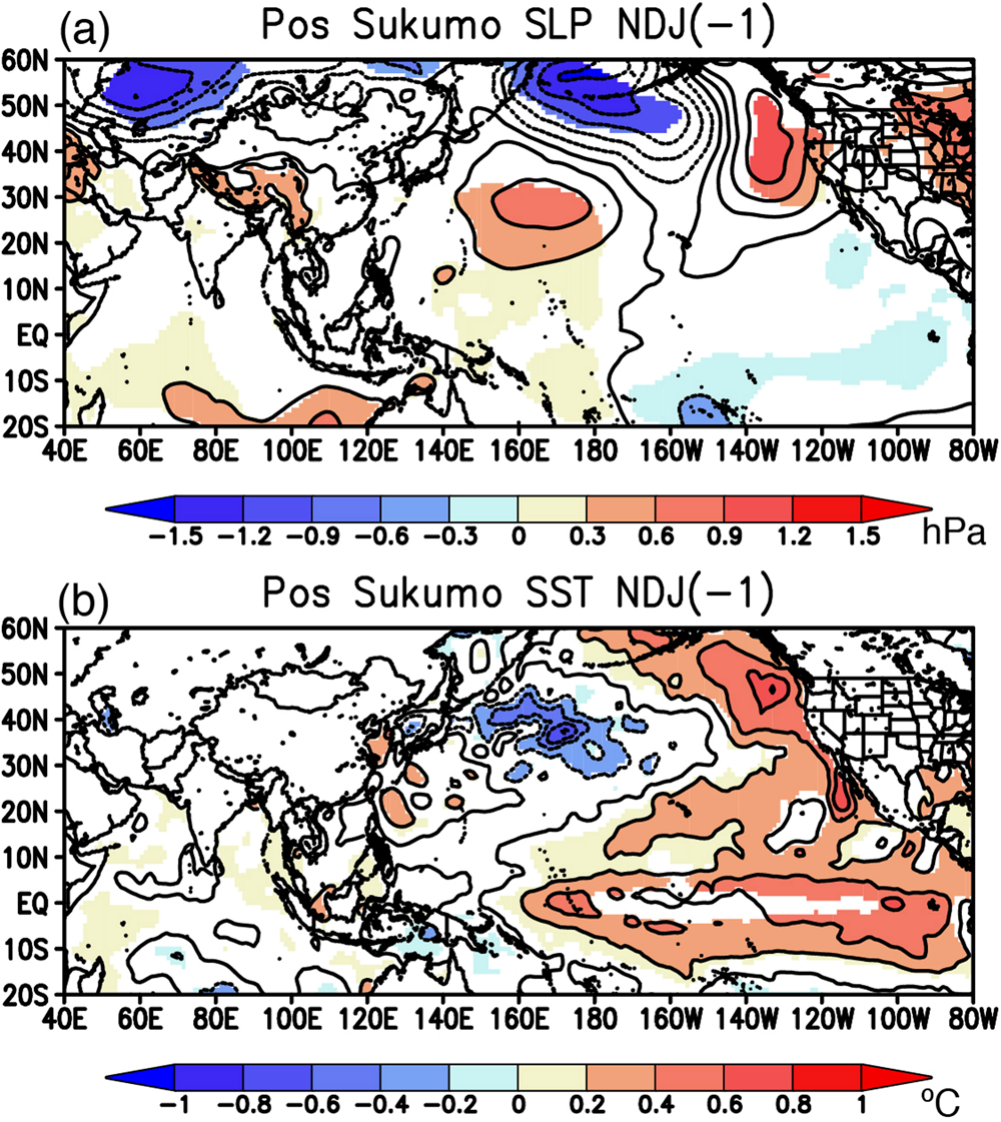
(**a**) Composite anomalies of November-January mean sea-level pressure (SLP, in hPa) one year before positive events (i.e. NDJ (-1)). Statistically significant anomalies exceeding 90% confidence level using a Student *t*-test are colored. (**b**) Same as in (**a**), but for the SST anomalies (in °C).

To explore the potential sources of the oceanic Rossby waves from the perspective of atmospheric forcing, we calculated composite anomalies with a one-year lag prior to the positive events for sea-level pressure (SLP) in Fig. 6a and surface wind stress curl in Fig. S5, respectively. The SLP anomalies exhibit significant positive values in the region of the Northwest Pacific (150–160°E at 30°N; Fig. 6a). This leads to anomalous anticyclonic wind stress curl that tends to induce downwelling oceanic Rossby waves, although the negative wind stress curl anomalies along 30°N show significant values in the very limited areas of 140–150°E (Fig. S5). The positive SLP anomalies in the Northwest Pacific are associated with negative sea-surface temperature (SST) anomalies to the northeast and positive SST anomalies to the southwest (Fig. 6b). These SST anomalies appear not to force the atmospheric variability, rather to be driven by the atmospheric forcing in such a way that anomalously dry northwesterly wind on the northeastern flank of the positive SLP anomalies in the Northwest Pacific increases evaporation and deepens the wintertime mixed layer, which enhances entrainment of cold water from deeper ocean. A similar but opposite process seems to operate for the positive SST anomalies on the southwestern flank of the positive SLP anomalies. Therefore, the SLP anomalies in the Northwest Pacific may be driven by remote forcing outside the Northwest Pacific, for example, atmospheric teleconnection from the tropical Pacific associated with El Niño-like condition (Fig. 6b), but potential sources of SLP anomalies require further detailed analysis and modelling studies.

## Discussions

This study has identified the potential roles of the Kuroshio variability on the wintertime fish resource variability estimated off Sukumo Bay and the remote influence from the atmospheric variability in the Northwest Pacific. Previous studies have attributed fish resource variability to regional variations in the physical and bio-geochemical conditions^4–6,16^, but little attention has been paid to its remote link with basin-scale ocean and atmosphere variability. The Kuroshio undergoes interannual variability under the influence of westward-propagating oceanic Rossby waves from the Northwest Pacific^19,21–23^. Since the Kuroshio variability is suggested to cause interannual modulation of small meanders off the southwest of Shikoku island^24^, the remotely induced oceanic Rossby waves have the potential to modulate the ocean circulation near the southwest coast of Japan and the amount of fish resource through changes in the subsurface ocean temperature. The identified relationship can be applied to other coastal regions along the western boundary currents in the subtropics where the westward-propagating oceanic Rossby waves may contribute to coastal ocean temperature variability^32^.

However, some issues behind the fish resource variability remain to be addressed. First, due to the limited availability of high-quality fish catch data (2006–2018), the established relationship between the fish resource and the ocean variables may be influenced by the outlier year, for example, 2016 with extremely high fish catch (Fig. 1a), but more prolonged datasets in the near future will help to verify the robustness of the statistical relationship. Second, the relationship between fish resource increase and anomalous warm water intrusion due to the northward approach of the Kuroshio may be straightforward, but due to the lack of coastal ocean observations and reanalysis results for bio-geochemical components, it is difficult to examine how the warm water affects the phyto- and zoo-plankton activities and the amount of fish species. Since the area off Sukumo Bay is largely influenced by nutrient-rich southward current from Bungo Channel during boreal winter, anomalous warm water advection from the Kuroshio into the channel may provide favorable temperature conditions for spawning grounds of small fishes such as sardines^33^ and enhance biomass and food availability for small larvae^34^. The warm water intrusion into Bungo Channel may be associated with more frequent occurrence of the Kyucho^25^, a coastal phenomenon with a sudden increase in coastal ocean current, but the Kyucho in Bungo Channel rarely occurs during boreal winter. Along this line, further observational and modelling studies involving the interaction of dynamical and bio-geochemical processes would advance our understanding of the fish resource variability off Sukumo Bay.

Previous studies have mainly focused on westward propagation of cyclonic oceanic Rossby waves and eddies along 30°N accompanied with the Kuroshio pathway variations southeast of Kyushu island^23,24^. On the other hand, the present study has also identified potential roles of westward-propagating anticyclonic eddies in the Kuroshio variations. The oceanic Rossby waves that influence the Kuroshio variability are not solely generated by atmospheric forcing but through internal ocean processes such as eddy-mean current interaction^31^. Since the subtropical Northwest Pacific at 30°N also receives influence of southward current associated with re-circulation from the Kuroshio extension current system (Fig. S6), the anticyclonic eddies traveling from the north may propagate westward via interaction with the re-circulation current. However, the relative contributions from the internal oceanic processes and the atmospheric forcing remain unclear. This needs to await further ocean modelling studies in which the atmospheric forcing such as wind stress curl is prescribed with or without interannual variations.

This study provides further implication for the development of fish resource prediction based on the ocean current information. Since there is a one-year lag relationship between anticyclonic eddies in the Northwest Pacific and anomalous increase in fish resource off Sukumo Bay, monitoring the SSH variability in the Northwest Pacific is imperative for predicting fish resource variability off Sukumo Bay one year ahead. Given that the SSH variability in the Northwest Pacific is driven mostly by the atmospheric variations, seasonal climate prediction over the Northwest Pacific using a global ocean-atmosphere coupled model may help extend the prediction lead time beyond one year. The long-term prediction information for fish resource would benefit fishery people to efficiently establish fishing plan as well as sustainably use and manage fish resources.

## Methodology

We analyzed daily fish catch data based on mid-size surrounding nets off Sukumo Bay, Shikoku island of Japan from 2006 to 2018. The data covers the period since 2006 when all the local fishery cooperatives near Sukumo Bay were integrated into the Sukumo Bay fishery cooperative. To estimate fish resource variability, we calculated the monthly fish catch per unit effort (CPUE) defined as the fish catch divided by the number of fishing days per person. For oceanic and atmospheric data over the global domain, we used the sea-surface temperature (SST) from the Optimum Interpolation SST version 2 (OISST V2)^35^ and the basic atmospheric variables from the ERA-Interim reanalysis results^36^. To estimate biological activity in the upper ocean, we used the monthly Chlorophyll-a data with 4-km horizontal resolution obtained from the Moderate Resolution Imaging Spectroradiometer (MODIS) on the Aqua satellite^37^. Here we analyzed all the above datasets with the same horizontal resolution of 1° × 1° over the domain.

To examine coastal ocean variability, we utilized monthly reanalysis results from the Japan Coastal Ocean Predictability Experiment 2 (JCOPE2)^38^ with a high horizontal resolution of 1/12° in the Northwest Pacific (108–180°E and 10.5–62°N). The JCOPE2 system is based on the Princeton Ocean Model (POM)^39^ with 46 vertical levels of sigma coordinate. The boundary condition of the JCOPE2 system is provided by relatively low-resolution model with a 1/4° horizontal resolution and 21 vertical levels in the entire Pacific. The low-resolution model was spun-up for 15 years using the monthly mean surface forcing from an initial condition with no motion, annual mean ocean temperature and salinity^40^. The spin-up results of the low-resolution model over the last five years are used for the lateral boundary condition of the high-resolution model, then both the low and high resolution models are integrated from Oct 1992 using surface wind stress, heat and salt fluxes of the six-hourly NCEP/NCAR reanalysis data^41^ via bulk formulae^42^. Besides atmospheric forcing, the ocean model benefited from data assimilation via three-dimensional variational assimilation (3DVAR)^43^ method using ocean observation data of sea-surface height (SSH) anomaly derived from several satellites, the SST from the Advanced Very High Resolution Radiometer/Multi-Channel SST (AVHRR/MCSST), and subsurface ocean temperature/salinity from the NOAA Global Temperature-Salinity Profile Program (GTSPP). Here we analyzed JCOPE2 reanalysis results during 1993–2018. To calculate monthly anomalies, we subtracted monthly climatology and removed a linear trend using a least squares method.

For the analysis of correlation between the fish resource (x) and the oceanic variables (y), we used Pearson product-moment correlation in which the least-squares regression line was calculated and the degree of the line fitting was evaluated using the least-squares method. Here, the correlation coefficient, R, was defined using the following equation:

1 $${\rm{R}}=\frac{\sum x{\text{'}}_{i}\,y{\text{'}}_{i}}{\sqrt{\sum x{\text{'}}_{i}^{2}}\sqrt{\sum y{\text{'}}_{i}^{2}}}$$

where the superscript’ of each variable means monthly detrended anomalies and the subscript i indicates the monthly time series.

## Supplementary information

Supplementary Figures file
