# Supplementary material for: Role of Kuroshio Current in fish resource variability off southwest Japan
Source: Sci Rep. 2019 Nov 29;9:17942. doi: 10.1038/s41598-019-54432-3 (PMC6884533; doi:10.1038/s41598-019-54432-3)
Supplement: Supplementary file 1 — Supplementary Figures file [file 41598_2019_54432_MOESM1_ESM.pdf]

# Supplementary information for
# "Role of Kuroshio Current in fish resource variability off southwest Japan"


Yushi Morioka[1], Sergey Varlamov[1], Yasumasa Miyazawa[1]

1: Application Laboratory, VAiG, JAMSTEC, Yokohama, Japan

Corresponding author: Dr. Yushi Morioka

E-mail: morioka@jamstec.go.jp


## Supplementary Figure Captions

**Figure S1:** Monthly lag autocorrelation of Nov-Jan mean CPUE off Sukumo Bay. Three-month mean CPUE are used to calculate the autocorrelation.

**Figure S2:** (a, b) Same as in Figs. 2c and d, but the correlation coefficients after removal of the outlier year 2016 associated with the extremely high CPUE. A black box indicates our region of interest off the south of Sukumo Bay. Hatched areas indicate correlation coefficients which are statistically significant above 80% confidence level of a Student *t*-test.

**Figure S3:** November-January mean Chlorophyll-a anomalies (in mg $m^{-3}$) averaged off the Sukumo Bay (black box in Fig. 2c) during 2006-2018.

**Figure S4:** (a) Vertical profile of November-January mean ocean temperature (in ºC) averaged off the Sukumo Bay. Black, red and blue lines correspond to a simple average of all the analysis years, positive and negative events defined in Fig. 3, respectively. (b) Same as in (a), but for the ocean density (in plus 1000 Kg $m^{-3}$).

**Figure S5:** Composite anomalies of November-January mean wind stress curl (in $10^{-7}$ N $m^{-3}$) one year before positive events. Statistically significant anomalies exceeding 90 % confidence level using a

Student *t*-test are colored. Negative values indicate anticyclonic circulation anomalies that tend to induce downwelling oceanic Rossby waves.

**Figure S6:** Same as in Fig. 4, but for seasonal mean SSH anomalies at 12-month lag (i.e. November-January one year before positive events) are shown, respectively. Statistically significant anomalies exceeding 90 % confidence level using a Student *t*-test are colored.

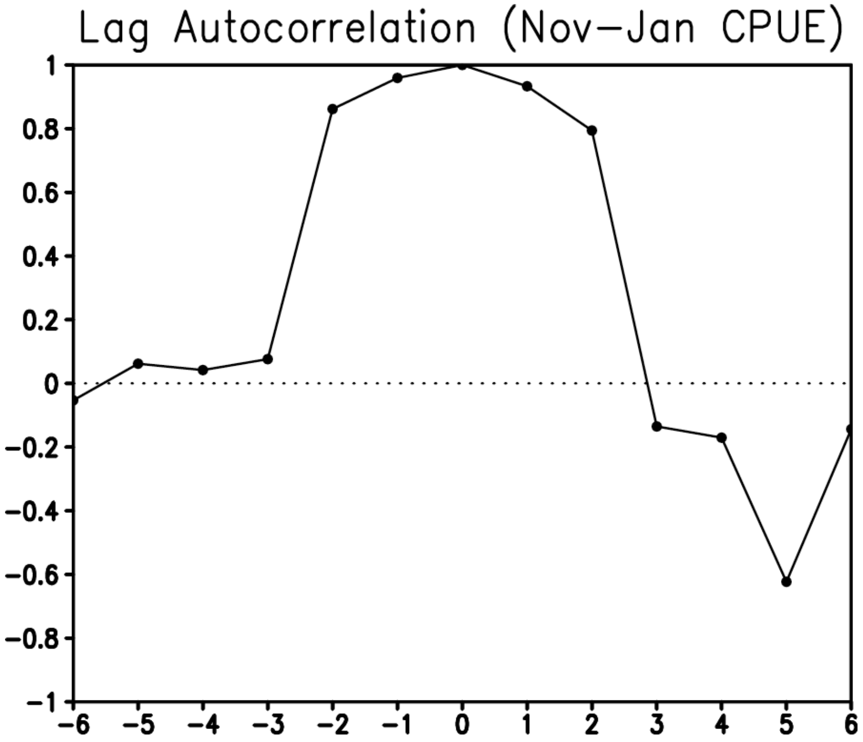


**Figure S1:** Monthly lag autocorrelation of Nov-Jan mean CPUE off Sukumo Bay. Three-month mean CPUE are used to calculate the autocorrelation.

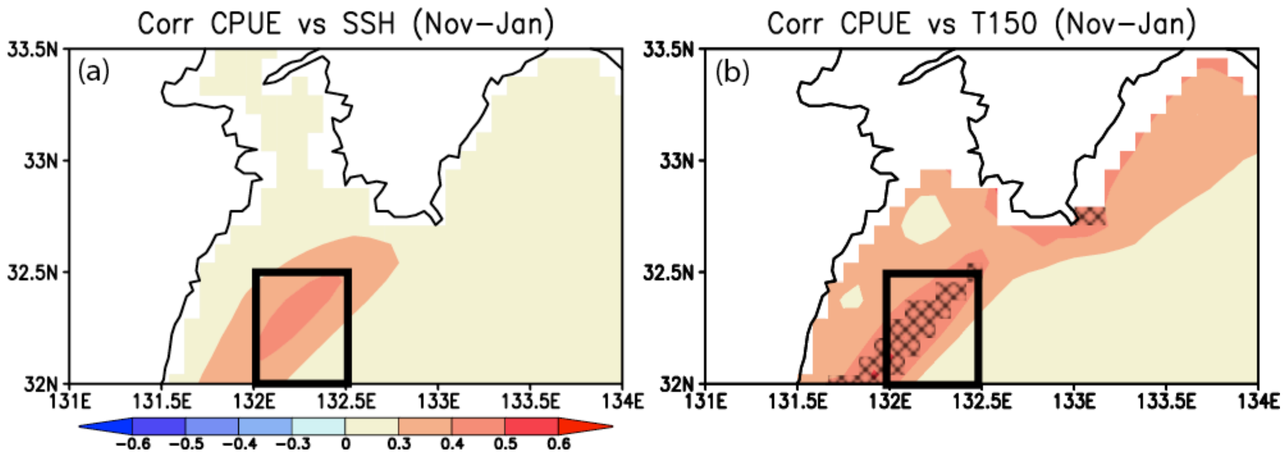


**Figure S2:** (a, b) Same as in Figs. 2c and d, but the correlation coefficients after removal of the outlier year 2016 associated with the extremely high CPUE. A black box indicates our region of interest off the south of Sukumo Bay. Hatched areas indicate correlation coefficients which are statistically significant above 80% confidence level of a Student *t*-test.

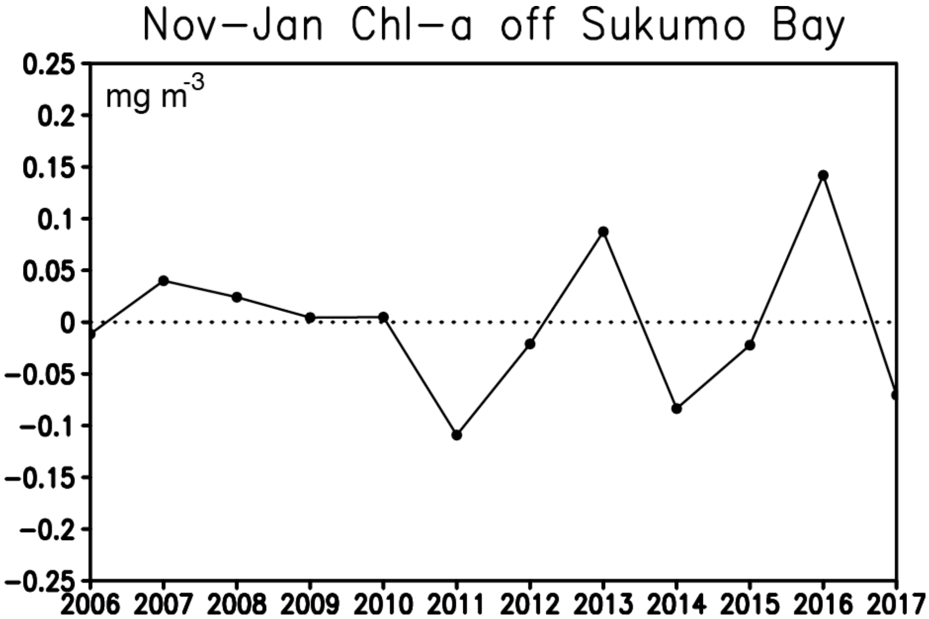


**Figure S3:** November-January mean Chlorophyll-a anomalies (in mg $m^{-3}$) averaged off the Sukumo Bay (black box in Fig. 2c) during 2006-2018.

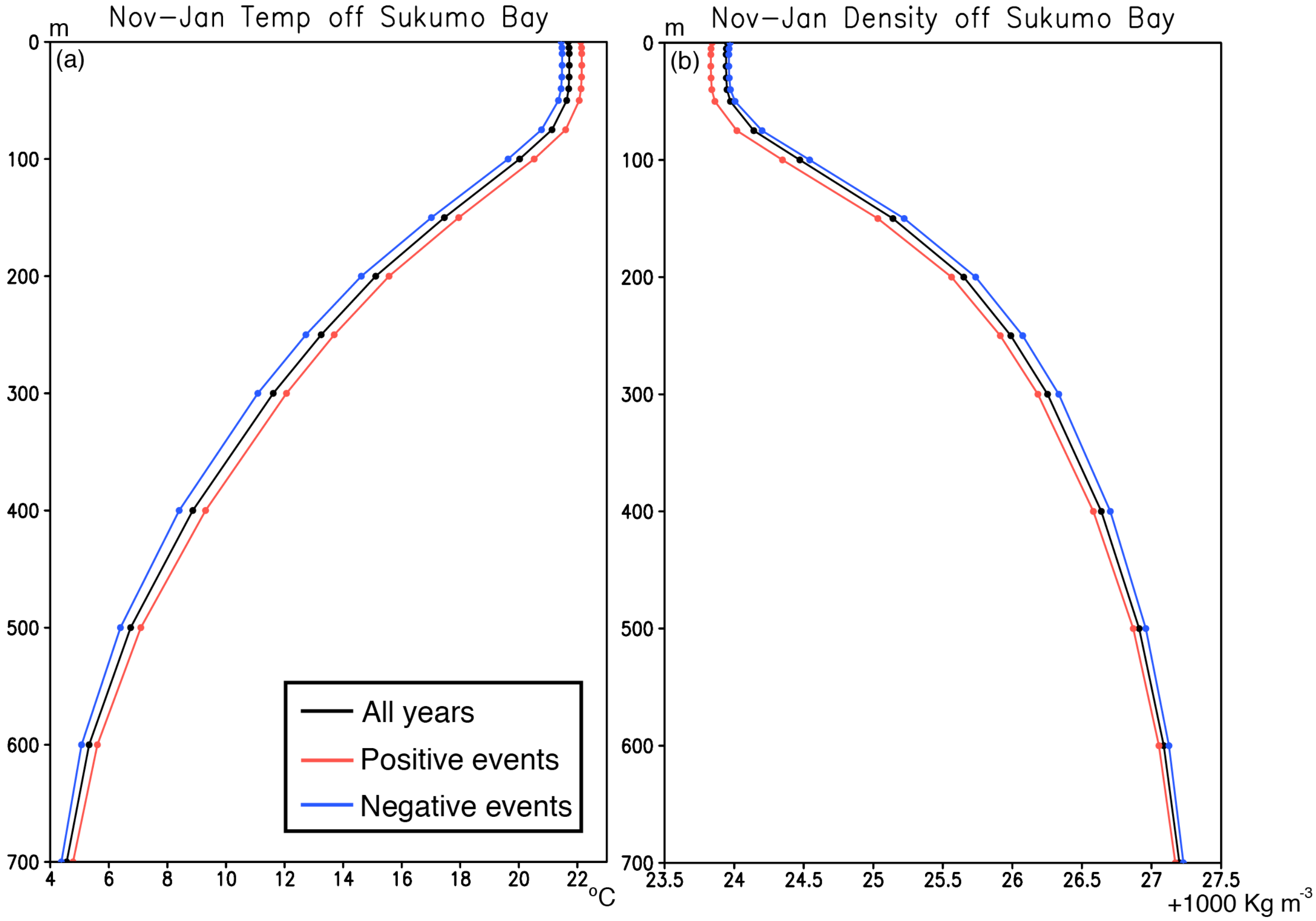


**Figure S4:** (a) Vertical profile of November-January mean ocean temperature (in °C) averaged off the Sukumo Bay. Black, red and blue lines correspond to a simple average of all the analysis years, positive and negative events defined in Fig. 3, respectively. (b) Same as in (a), but for the ocean density (in plus 1000 Kg $m^{-3}$).

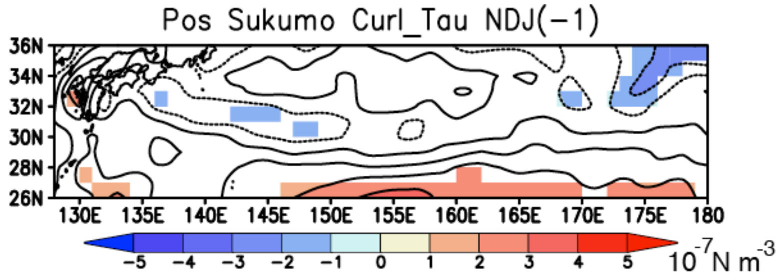


**Figure S5:** Composite anomalies of November-January mean wind stress curl (in $10^{-7}$ N $m^{-3}$) one year before positive events. Statistically significant anomalies exceeding 90 % confidence level using a Student *t*-test are colored. Negative values indicate anticyclonic circulation anomalies that tend to induce downwelling oceanic Rossby waves.

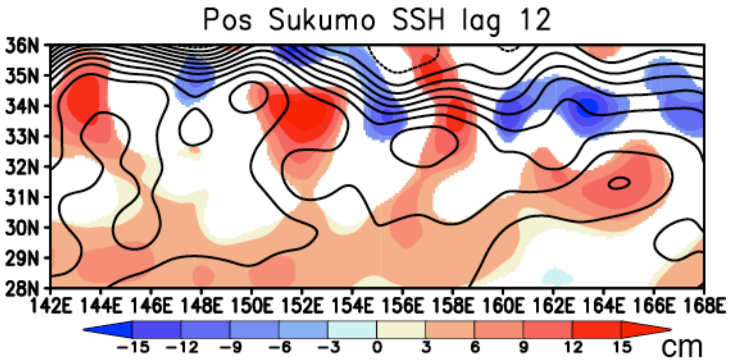


**Figure S6:** Same as in Fig. 4, but for seasonal mean SSH anomalies at 12-month lag (i.e. November-January one year before positive events) are shown, respectively. Statistically significant anomalies exceeding 90 % confidence level using a Student *t*-test are colored.
